# Central transthyretin acts to decrease food intake and body weight

**DOI:** 10.1038/srep24238

**Published:** 2016-04-07

**Authors:** Fenping Zheng, Yonwook J. Kim, Timothy H. Moran, Hong Li, Sheng Bi

**Affiliations:** 1 Department of Psychiatry and Behavioral Sciences, Johns Hopkins University School of Medicine, Baltimore, Maryland 21205, USA; 2 Department of Endocrinology, The Sir Run Run Shaw Hospital, Zhejiang University School of Medicine, Hangzhou 310016, China

## Abstract

Transthyretin (TTR) is a blood and cerebrospinal fluid transporter of thyroxine and retinol. Gene expression profiling revealed an elevation of *Ttr* expression in the dorsomedial hypothalamus (DMH) of rats with exercise-induced anorexia, implying that central TTR may also play a functional role in modulating food intake and energy balance. To test this hypothesis, we have examined the effects of brain TTR on food intake and body weight and have further determined hypothalamic signaling that may underlie its feeding effect in rats. We found that intracerebroventricular (icv) administration of TTR in normal growing rats decreased food intake and body weight. This effect was not due to sickness as icv TTR did not cause a conditioned taste aversion. ICV TTR decreased neuropeptide Y (NPY) levels in the DMH and the paraventricular nucleus (*P* < 0.05). Chronic icv infusion of TTR in Otsuka Long-Evans Tokushima Fatty rats reversed hyperphagia and obesity and reduced DMH NPY levels. Overall, these results demonstrate a previously unknown anorectic action of central TTR in the control of energy balance, providing a potential novel target for treating obesity and its comorbidities.

Obesity has become a public health problem and has been linked to various life-threatening diseases such as cardiovascular disease and type 2 diabetes. The body maintains a healthy weight through regulating food intake and energy expenditure. Both overeating and sedentary lifestyle contribute to the development of obesity, whereas increased physical activity or exercise improves weight loss and, when added to dietary control, becomes a key factor for success in long-term weight maintenance in previously overweight and obese individuals^1^. Recent work with rodent obesity models has shown that exercise affects body weight and body composition not only by increasing energy expenditure, but also by altering appetite and reducing food intake^2^,^3^,^4^. Human studies have also shown that intense exercise reduces daily energy balance in adolescents with obesity by both increasing energy expenditure and decreasing food consumption^5^. Despite these compelling observations, the neural/molecular mechanisms underlying these effects remain largely unknown.

The hypothalamus is a brain region that integrates central and peripheral signals of energy status to modulate food intake and energy expenditure to maintain energy homeostasis^6^,^7^. We have recently demonstrated the importance of dorsomedial hypothalamic (DMH) neural signaling in the mediation of the effects of exercise on energy balance^3^,^8^. Otsuka Long-Evans Tokushima Fatty (OLETF) rats are a rodent model of obesity and diabetes^9^,^10^. We have identified elevated expression of the orexigenic peptide neuropeptide Y (NPY) in the DMH of OLETF rats and have indicated that this elevation contributes to their obesity and diabetes^11^,^12^,^13^. Furthermore, we have demonstrated that exercise reversed the hyperphagia and obesity of OLETF rats^3^. Together, these results indicate that exercise limits the orexigenic effects of DMH NPY in OLETF rats, but the molecule(s) mediating these effects remain to be determined^14^.

To search for such molecule(s), we have examined gene expression profiling in the DMH of rats in response to running wheel access using a DNA microarray analysis. We have found that transthyretin (TTR) expression was significantly increased in the DMH of rats with exercise-induced reductions in food intake and body weight gain. We now know that TTR is a blood and cerebrospinal fluid (CSF) transporter of thyroxine and retinol^15^. Data have also shown that TTR plays an important role in amyloidosis^16^,^17^ and in the prevention of Alzheimer’s disease^18^,^19^. Although TTR has often been used as a nutritional biomarker^20^, its role in the control of energy balance has yet to be established. Elevation of TTR in the DMH of rats with exercise-induced anorexia implies that DMH or central TTR may also play a functional role in modulating food intake and energy balance. Therefore, we have conducted follow-up studies to test this potential role. We have examined the effects of intracerebroventricular (icv) administration of TTR on food intake and body weight in rats and assessed the potential neural mechanisms underlying these effects. Herein, we report that central TTR acts as an anorectic to lower body weight via, at least in port, decreasing hypothalamic NPY signals.

## Results

### DMH TTR elevation in rats with exercise-induced anorexia

**Figure 1:**
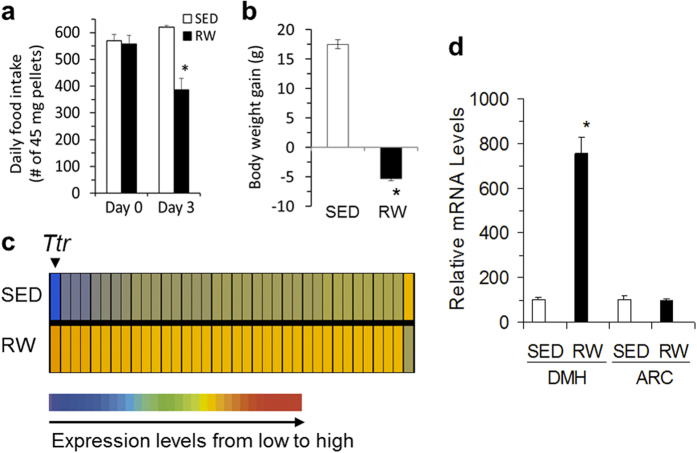
Exercise-induced elevation of *Ttr* expression in the dorsomedial hypothalamus (DMH). (**a,b**) Rats with voluntary access to running wheels (RW) for 3 days had decreased food intake (**a**) and body weight (**b**) compared to sedentary rats (SED). (**c**) Microarray analysis of rat genome shows that using criterion of a 2-fold change, 36 out of the total 31,099 genes were identified in the DMH of RW rats relative to SED controls (an arrowhead indicates a great increase in *Ttr* gene expression). The 36 genes affected are listed in Table 1. (**b**) Real-time RT-PCR confirmed a more than 7-fold increase in *Ttr* expression in the DMH, but unaltered expression in the arcuate nucleus (ARC) of RW rats. Values are means ± SEM. n = 6 rats per groups. *P < 0.05 vs. SED rats analyzed using Student’s t test (two-tailed).

Three-day voluntary access to running wheels (RW) resulted in significant decreases in food intake and body weight gain in rats (*P* < 0.05, Fig. 1a,b). As shown in Fig. 1a,b, RW rats ate 40% less food than sedentary rats (SED) and while SED rats gained 18 gram in three days, RW rats loss 5 gram. Following three-day RW access, we profiled gene expression in the DMH, and identified an induction of *Ttr* expression in the DMH of RW rats (Fig. 1c & Table 1). Real-time reverse transcription polymerase chain reaction (RT-PCR) confirmed a significant increase in *Ttr* expression in the DMH of RW rats relative to that of SED rats (*P* < 0.05), whereas *Ttr* expression in the arcuate nucleus (ARC) did not differ between these two groups (Fig. 1d).

**Figure 2:**
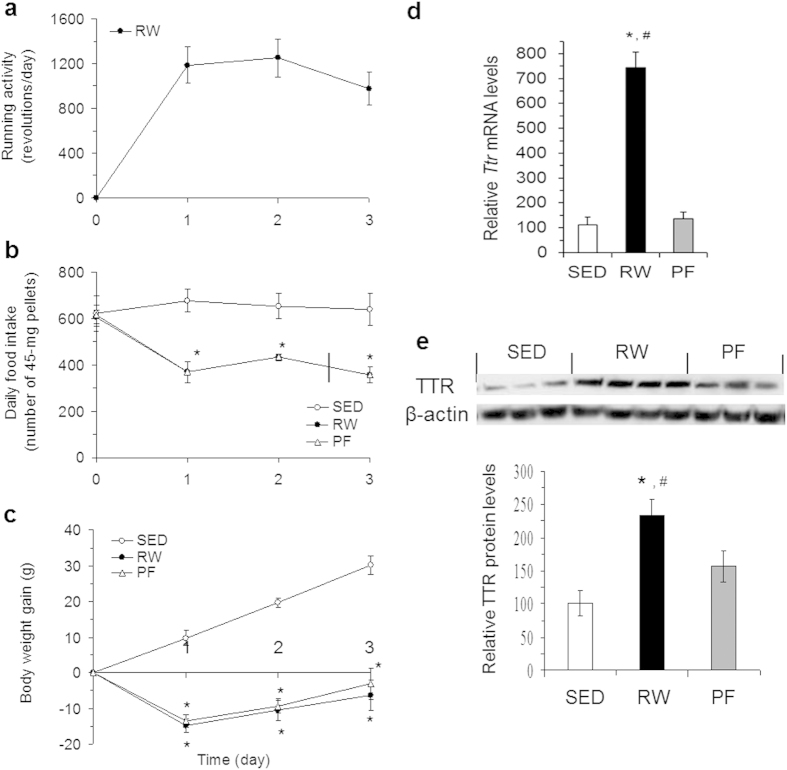
Effects of exercise on DMH TTR. (**a**) Running activity (one revolution ≈ 1 meter) in rats with voluntary access to RW for 3 days. (**b**) Food intake in the three groups of SED, RW, and pair-fed (PF) rats. (**c**) Body weight gain in the three groups. (**d**) Three days later, *Ttr* mRNA expression in the DMH was determined using real-time RT-PCR. (**e**) TTR levels in the DMH were examined using Western blot with anti-TTR antibody. Values are means ± SEM. *n* = 6–8 rats. *P < 0.05 vs. SED rats and ^#^*P* < 0.05 vs. PF rats determined by one-way analysis of variance (ANOVA), followed by pairwise multiple Fisher’s LSD comparisons.

To assess whether DMH *Ttr* elevation is a primary cause of exercise or a secondary response to reductions of food intake and body weight, we examined *Ttr* expression in the DMH of pair-fed (PF) rats. As expected, running wheel access (or exercise) significantly decreased food intake, and PF rats consumed the same amount of food as RW rats (Fig. 2a,b). Both of them had equal reductions in body weight (Fig. 2c), indicating that weight loss produced by running activity results primarily from decreased food intake. Subsequent determination of DMH *Ttr* expression revealed that mRNA levels of *Ttr* were significantly increased in the DMH of RW rats but not in PF rats (Fig. 2d). This provides support for the view that exercise causes elevated expression of *Ttr* in the DMH. Consistent with this change, TTR protein levels were also increased in the DMH of RW rats. As shown in Fig. 2e, Western blot analysis revealed that while DMH TTR was weakly detected in SED rats, its levels were significantly increased in RW rats relative to both SED and PF rats. In contrast, DMH TTR levels did not differ significantly between PF and SED rats (Fig. 2e). Together, these results underscore the potential contribution of DMH TTR elevation to exercise-induced reductions in food intake and body weight gain, suggesting that DMH or central TTR may act as an anorectic to affect energy balance.

**Figure 3:**
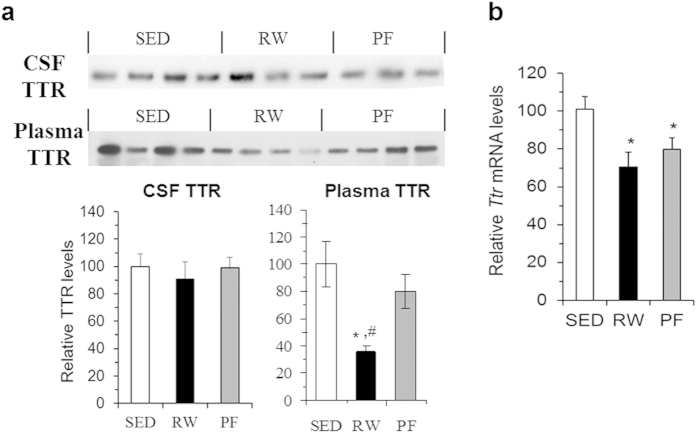
Effects of exercise on plasma and CSF TTR levels and liver mRNA expression. (**a**) Following three-day access to WR, TTR levels in the CSF and plasma were examined using Western blot with anti-TTR antibody. Ten microliters of CSF or plasma were loaded. (**b**) *Ttr* mRNA expression in liver was determined using real-time RT-PCR. Values are means ± SEM. *n* = 6–8 rats. *P < 0.05 vs. SED rats and ^#^*P* < 0.05 vs. PF rats determined by one-way ANOVA, followed by pairwise multiple Fisher’s LSD comparisons.

Given that TTR is a circulating and CSF protein and is mainly produced from liver and choroid plexus respectively^15^, we measured blood and CSF TTR levels as well as examined its expression in the liver and choroid plexus. As shown in Fig. 3a, TTR levels were unchanged in the CSF, but significantly decreased in the plasma of RW rats. Consistent with these observations, *Ttr* expression in the choroid plexus did not differ among the three groups of rats (data not shown). Levels of *Ttr* expression were significantly decreased in the liver of RW rats compared to that of SED rats, but did not differ between RW and PF rats (Fig. 3b). Thus, these results indicate that exercise has differential effects on brain and peripheral TTR.

### Central TTR affects food intake and body weight

**Figure 4:**
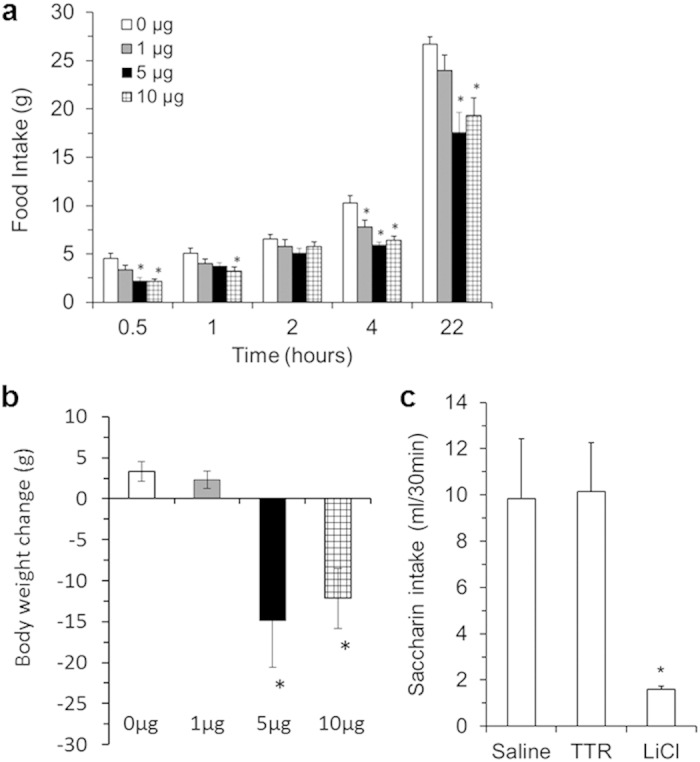
Effects of central TTR on food intake and body weight. (**a**) Rats received 0, 1, 5, or 10 μg of intracerebroventricular (icv) TTR just before lights-off and food intake was examined at 0.5, 1, 2, 4, and 22 hours after icv injection (*n* = 8–10 rats). Data were analyzed using two-way repeated-measures ANOVA. (**b**) Body weight change was determined by weight before and 22 hours after TTR injection (*n* = 8–10 rats, one-way ANOVA). (**c**) A conditioned taste aversion (CTA) test in an additional cohort. The subsequent intake of 0.15% saccharin solution was examined in saline control (Saline), icv TTR (TTR), and positive control (LiCl) rats (n = 5–6 rats, one-way ANOVA). Values are means ± SEM. *P < 0.05 vs. Saline rats.

To test this concept, we examined whether centrally administered TTR affects food intake and body weight in normal growing rats. As shown in Fig. 4a, icv administration of TTR resulted in a significant reduction of food intake. While a low dose of TTR (1 μg) reduced food intake at 4 hours post-injection, a higher dose of TTR (5 μg) inhibited food intake over 22 hours (Fig. 4a). ICV TTR at 10 μg did not further reduce 22-hour food intake, suggesting that we has reached a ceiling action with a 5 μg dose of TTR, except that 10 μg TTR also reduced food intake at 1 hour post-injection (Fig. 4a). Both 5 and 10 μg of icv TTR caused significant weight loss (Fig. 4b).

### Central TTR does not induce visceral illness

Because it is possible that the anorectic effect of icv TTR was due to TTR-induced visceral illness, we next conducted a conditioned taste aversion (CTA) test to assess this possibility. As shown in Fig. 4c, the subsequent intake of 0.15% saccharin solution previously paired with TTR administration was not affected, indicating that icv TTR did not cause a CTA or visceral illness, whereas the conditioned treatment with the positive control agent LiCl resulted in a significant reduction of subsequent saccharin intake, indicating development of CTA in these animals.

### TTR reduces hypothalamic NPY levels

**Figure 5:**
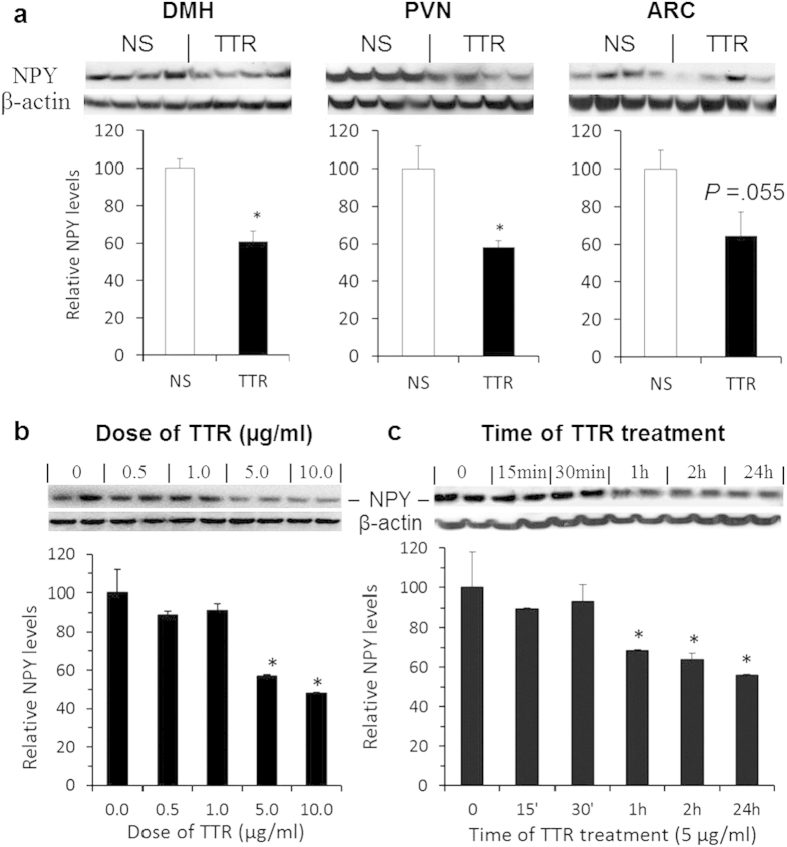
Effects of TTR on hypothalamic NPY levels. (**a**) Two-hour fasted rats received icv administration of saline (NS) or 10 μg TTR (TTR). One hour post-injection, rats under a fasting condition were euthanized, and NPY levels in the DMH, the PVN, and the ARC were examined using Western blot with anti-NPY antibody and analyzed by Student’s *t*-test (*n* = 4–6 rats). *P < 0.05 vs. saline-treated rats. (**b,c**) *In vitro* primary hypothalamic neuron cultures, dose (**b**) and time course (**c**) effects of TTR on NPY levels in primary neuronal cells determined by Western blot with anti-NPY antibody. *P < 0.05 vs. a control group (0 dose or time point) analyzed using one-way ANOVA. Anti-β-actin antibody was used for a loading control.

To investigate the neural mechanism of TTR’s action, we examined whether centrally administered TTR alters hypothalamic NPY signals as a previous report has shown that TTR has a proteolytic effect on NPY^21^. Within the hypothalamus, NPY-producing neurons are primarily found in the ARC and the DMH^22^ and the PVN serves as a main output of the hypothalamus to modulate food intake and body weight^23^,^24^,^25^. We examined NPY levels specifically in these three nuclei in rats receiving icv TTR. We found that icv administration of TTR resulted in significant decreases in NPY levels in the DMH and the PVN (*P* < 0.05), and a trend for decreases in the ARC (*P* = 0.055, Fig. 5a). We further verified the effect of TTR on NPY levels using *in vitro* primary hypothalamic neuron cultures, mimicking the *in vivo* hypothalamic effect of TTR. NPY levels were significantly decreased by TTR treatment at both doses of 5 and 10 μg/ml (Fig. 5b). This effect started after 1 hour of TTR treatment and lasted over 24 hours (Fig. 5c).

**Figure 6:**
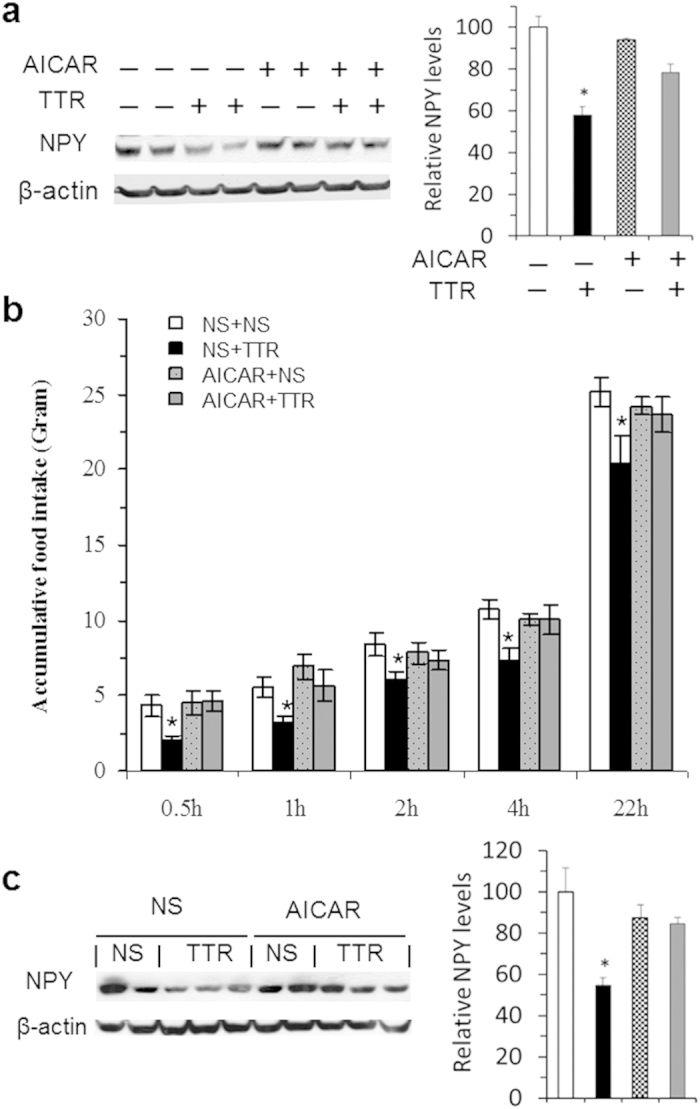
Anorectic effect of TTR via hypothalamic NPY. (**a**) Pre-treatment with the AMPK activator AICAR attenuated TTR-induced reduction of NPY levels in primary neuronal cells. Cells were treated with control PBS & PBS (−, −), PBS & 10 μg TTR (−, +), 20 μM AICAR & PBS (+, −), or 20 μM AICAR & 10 μg TTR (+, +). AICAR was added 1 hour before TTR treatment. After 2 hours of TTR treatment, cells were harvested and lysed. NPY levels were determined using Western blot with anti-NPY antibodies. (**b**) Two-hour fasting rats were pre-treated with icv saline (NS) or AICAR (300 μg) one hour prior to icv NS or TTR (10 μg) that were given just before lights-off, designated as NS+NS, NS+TTR, AICAR+NS, and AICAR+TTR. Mean (+ SEM) food intake was examined at 0.5, 1, 2, 4, and 22 hours after the second injection, and analyzed using three-factors ANOVA with one repeated-measures (n = 7 rats). (**c**) After feeding test and recovery, rats received the same treatments as above, but chow was not returned. One hour post-second injection, mean (±SEM) NPY levels in the PVN were determined using Western blot and analyzed using two-way ANOVA (n = 3–5 rats). *P < 0.05 vs. NS+NS controls.

Evidence has indicated that increased activity of AMP-kinase (AMPK) promotes hypothalamic NPY products^26^. We next assessed whether TTR-induced reductions of NPY levels and food intake can be prevented by pre-treatment with the AMPK activator. We first tested this idea in an *in vitro* study. Indeed, we found that while TTR treatment resulted in decreased NPY levels in hypothalamic neuronal cultures, this decrease was attenuated by pre-treatment with the AMPK activator AICAR (Fig. 6a). A follow-up *in vivo* study further revealed that while food intake was significantly reduced in rats receiving icv TTR, pre-treatment with icv AICAR prevented this reduction (Fig. 6b). Moreover, pre-treatment with AICAR prevented TTR-induced reduction of NPY levels in the PVN, a main projection site of hypothalamic NPY neurons (Fig. 6c). Together, these results demonstrate that central TTR is capable of affecting hypothalamic NPY levels to modulate food intake.

### Central TTR reverses hyperphagia and obesity of OLETF rats

**Figure 7:**
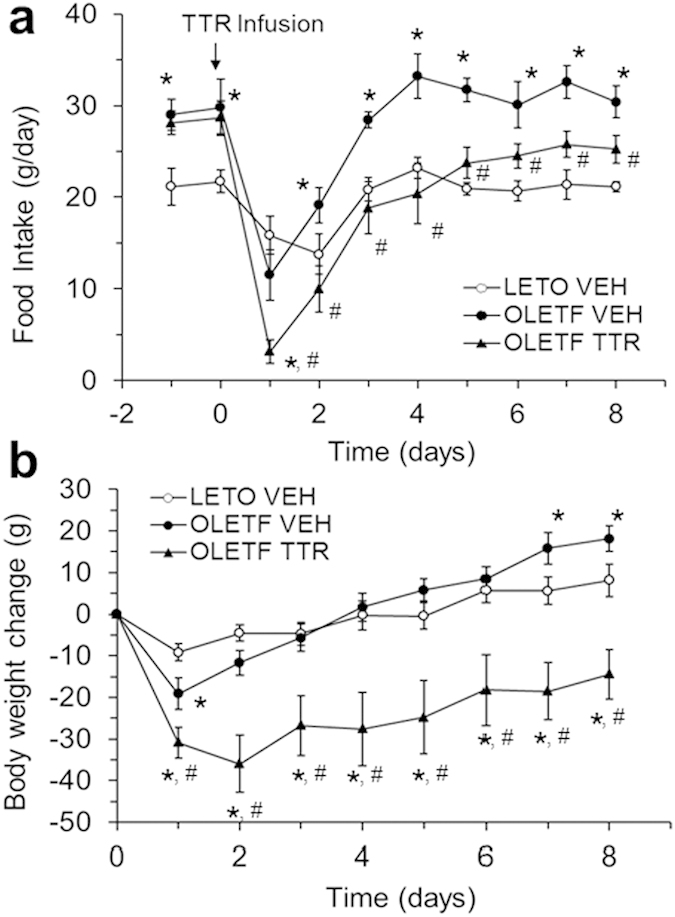
Effects of central TTR on hyperphagia and obesity. OLETF and lean LETO rats were infused with icv saline (VEH) or TTR (10 μg/day) via mini-osmotic pumps for 8 days. (**a**) Food intake and (**b**) body weight change were examined over 8 days and analyzed using two-way repeated-measures ANOVA (*n* = 7–10 rats). Values are means ± SEM. *P < 0.05 vs. LETO VEH and ^#^*P* < 0.05 vs. OLETF VEH.

Consistent with exercise-induced elevation of TTR in the DMH, we also identified an elevation of *Ttr* expression in the DMH of exercised OLETF rats (data not shown). A previous report has shown that exercise reverses hyperphagia and obesity of OLETF rats^3^. We next tested whether chronic icv infusion of TTR into OLETF rats can produce similar reversal effects on hyperphagia and obesity as seen in exercise OLETF rats. Prior to TTR treatment, OLETF rats at 18 weeks of age ate significantly more food (by 37% higher, Fig. 7a) and were significantly heavier (544 ± 10 g in OLETF vs. 424 ± 12 g in LETO rats, by 28% greater) than lean Long-Evans Tokushima Otsuka (LETO) rats, indicating that OLETF rats became hyperphagic and obese. TTR infusion resulted in significant reductions of both food intake (Fig. 7a) and body weight gain in OLETF rats (Fig. 7b). While vehicle-treated OLETF rats continued to have significantly increased food intake by about 142% of that consumed by LETO rats, OLETF rats with TTR treatment reduced their intake to a level similar to that of LETO rats over 8 days (Fig. 7a). At the end of infusion, vehicle-treated OLETF rats gained 18 grams, whereas OLETF rats with TTR treatment lost 14 grams of body weight (Fig. 7b). As expected, Western blot revealed that NPY levels in the DMH of OLETF rats receiving icv TTR were significantly decreased by 41% relative to those of vehicle-treated OLETF rats (59 ± 8 in OLETF TTR vs. 100 ± 13 in OLETF VEH, *P* < 0.05).

## Discussion

In the present study, we have unmasked a previously unknown role for central TTR in modulating food intake and body weight. Our gene expression profiling revealed a significant elevation of TTR in the DMH of rats with exercise-induced reductions in food intake and body weight gain. This finding led us to propose that central TTR plays an important role in the control of food intake and energy balance. Indeed, we found that icv TTR inhibited food intake and body weight. This treatment reduced hypothalamic NPY levels. Furthermore, chronic icv infusion of TTR into OLETF rats lowered DMH NPY levels and ameliorated hyperphagia and obesity. Overall, these results demonstrate that central TTR acts as an anorectic to affect energy balance.

NPY is a potent hypothalamic orexigenic peptide^22^. Within the hypothalamus, ARC NPY has been shown to play an important role in the regulation of energy balance. ARC NPY neurons contain leptin receptors (LepRb) and serve as one of the downstream mediators of the anorectic actions of leptin^6^,^7^. Physiologically, *Npy* gene expression in the ARC is increased in response to food deprivation^27^,^28^,^29^, but is decreased in the state of overeating or overweight^30^ in order to maintain energy homeostasis. Consistent with this notion, while exercise causes a negative effect on energy balance via increasing energy expenditure and/or decreasing food intake, exercise results in increased expression of *Npy* in the ARC^3^. This suggests that in response to exercise, ARC NPY system is up-regulated to meet exercise-induced increase in energy demands in order to restore energy balance.

NPY-expressing neurons have also been found in the DMH^22^,^27^, although the critical mechanism of DMH NPY’s action in energy balance control remains incompletely understood. We have investigated the neural mechanism underlying the development of hyperphagia and obesity of OLETF rats that congenitally lack cholecystokinin (CCK)-1 receptors^31^ and have found *Npy* overexpression specifically in the DMH of both pair-fed, normal weight adult and pre-obese young OLETF rats. In contrast, we have found no primary deficit in ARC NPY regulation as ARC NPY is down-regulated in obese and normalized in pair-fed OLETF rats^11^,^13^. These data suggest that DMH NPY overexpression causes hyperphagia and obesity of OLETF rats. Our follow-up studies provide support for this view by demonstrating that knockdown of NPY in the DMH of OLETF rats via adeno-associated virus (AAV)-mediated RNAi ameliorates these alterations^12^. We now appreciate that in contrast to ARC NPY, DMH NPY neurons do not co-express LepRb and their actions are not under the control of leptin^29^. In fact, DMH NPY neurons contain CCK-1 receptors and parenchymal microinjection of CCK into the DMH inhibits DMH *Npy* expression and reduces food intake^32^,^33^. These data indicate that DMH NPY exerts a distinct action in the control of food intake and body weight^22^. Furthermore, previous studies have shown that exercise reverses hyperphagia and obesity and causes down-regulation of DMH NPY in OLETF rats^3^,^14^, indicating that exercise produces a suppressive effect on DMH NPY action in OLETF rats independently of DMH CCK. This implies that other molecule(s) may mediate the reversal effects of exercise in OLETF rats.

The present study is aimed at exploring such molecule(s). We found that (1) exercise increased levels of TTR specifically in the DMH, (2) icv administration of TTR reduced food intake and body weight and decreased hypothalamic NPY levels, (3) preventing TTR-induced reduction of NPY prevented its anorectic effect, and (4) mimicking the effect of exercise in OLETF rats, chronic icv infusion of TTR in OLETF rats reduced DMH NPY levels and reversed their hyperphagia and obesity. The finding of the suppressive effect of TTR on NPY levels is consistent with a previous report that TTR knockout results in increased levels of NPY in the central and peripheral nervous systems, although whether or not NPY levels were altered in the hypothalamus of TTR knockout mice was not examined in that report^34^. In addition, a previous report has shown that although TTR knockout did not affect overall chow intake and body weight, this knockout causes increased carbohydrate consumption and preference^34^, implying that TTR is involved in modulation of feeding behavior. Our present results provide support for this view and further demonstrate that central TTR can act as a neuromodulator in the regulation of food intake and energy balance, at least in part, via affecting hypothalamic NPY signals.

It is worth addressing that exercise increases the renal clearance of TTR (by more than 8 times)^35^, which contributes to lowered blood TTR levels. Our findings of decreased levels of blood TTR in exercised rats are consistent with this previous report. Importantly, data have shown that blood TTR determines the clearance of circulating retinol-binding protein 4 (RBP4) and high levels of RBP4 contribute to insulin resistance^36^. We envisage that while exercise affects central and peripheral TTR levels in an opposite direction, its resulting changes actually improve both energy and glucose homeostasis. Thus, the detailed effect of exercise on peripheral TTR also merits further investigation.

Various evidence has shown that dissociation, misfolding and aggregation of wild type TTR tetramer are associated with senile systemic amyloidosis (SSA) and mutations of the *Ttr* gene facilitate more facile dissociation and/or misfolding and amyloidogenesis^37^. Despite such linkages, molecular insights into the onset and progression of amyloid diseases remain largely unclear. In fact, previous reports have shown that TTR can sequester amyloid beta protein and prevent amyloid formation in Alzheimer’s disease^18^,^19^. Thus, it is also worth further evaluating the influence of TTR on energy homeostatic regulation and amyloidogenesis. The outcomes from such studies will provide important evidence directing us to design better strategies to treat obesity and amyloidosis.

In summary, we have identified an important role for central TTR in the control of food intake and body weight. Particularly, TTR can be produced in the DMH in response to exercise, and within the hypothalamus, TTR serves as an anorectic to modulate food intake and energy balance, at least in part, via affecting hypothalamic NPY levels. Together, these results not only establish a novel function of brain TTR, but also provide a potential target for the prevention and treatment of obesity and its comorbidities.

## Methods

### Animals

Male Sprague-Dawley rats weighing 250–275 g were purchased from Charles River Laboratories (Wilmington, MA, USA). Rats were individually housed under a 12:12-hour light–dark cycle (lights on at 0100 hours) in a temperature-controlled environment (22 °C ~ 24 °C) with ad libitum access to water and standard regular chow, except where noted. All procedures were approved by the Institutional Animal Care and Use Committee at Johns Hopkins University and in accordance with the National Institute of Health’s Guide for the Care and Use of Laboratory Animals.

### Running activity, food intake, and body weight

In a microarray study, 12 Sprague-Dawley rats were transferred to running wheel cages containing computerized pellet dispensers (MED Associates), which delivered 45-mg chow pellets (F0165, Bio-Serv., Frenchtown, NJ) in response to removal of the previous pellet and each delivery was computer monitored^8^. Running wheels were initially locked for 7 days of environmental familiarization. After habituation, rats were weight-matched and randomly divided into two groups (*n* = 6 rats): a sedentary group (SED) continued to be kept in cages with a locked running wheel and an exercise group had voluntary access to running wheels (RW) for 3 days. All rats had ad lib access to chow pellets. Running activity and food intake were computer-monitored 24 hours daily. Body weight was measured daily. After 3 days, rats were decapitated under isoflurane anesthesia, and brains were harvested for subsequent microarray analyses of gene expression in the DMH. In the second experiment, 24 rats were used. As described above, after habituation rats were randomly divided into three groups (*n* = 8 rats): one sedentary group, one exercise group, and one additional group of pair-fed rats (PF) in which rats were kept in cages with a locked running wheel but their pellet dispensers were yoked to individual dispensers of exercise rats, so that pair-fed rats had the same amount and pattern of food intake as did exercise rats. Running activity, food intake and body weight were recorded as above. Three days later, rats were anesthetized under a mixture of ketamine (100 mg/kg) and xylazine (20 mg/kg) intraperitoneally (ip). CSF (free of blood contamination) was obtained by cisternal puncture. Following CSF sampling, rats were decapitated, trunk blood was collected, liver was sampled, and brains were saved for subsequent determinations of TTR protein or mRNA expression levels using Western blot or real-time RT-PCR analysis.

### Microarray analysis

The DMH and the arcuate nucleus (ARC)^38^ were punched out and total RNA from each sample was extracted using a RNeasy Mini kit (Qiagen). A microarray assay of gene expression in the DMH was conducted in the Johns Hopkins Microarray Core Facility. Briefly, samples were labeled with biotin, hybridized to commercial Affymatrix GeneChip Array (Rat Genome 230 2.0 Array), and detected with Streptavidin-phycoerythrin Biotinylated anti-streptavidin antibody. Microarray data were analyzed with GeneSpring software (GeneSpring GX 7.3, Agilent Technologies Inc.). Real-time RT-PCR was conducted to verify *Ttr* expression in the DMH and the ARC.

### Central injection of TTR

Sprague-Dawley rats were individually housed and unilaterally implanted with chronic indwelling lateral ventricular cannulas as described previously^8^. Briefly, rats were anesthetized with a mixture of ketamine (100 mg/kg) and xylazine (20 mg/kg) ip, and placed in a stereotaxic device. A 23-gauge stainless steel guide cannula (Plastics One, Roanoke, VA) was implanted 5.0 mm below the dura, 1 mm caudal to the bregma, and 1.3 mm lateral to the midline^38^. A 30-gauge stainless steel obturator (Plastics One) was inserted into the cannula to maintain potency. Following a week of postsurgical recovery, rats were given pseudo-injections during the measurement of daily body weight, i.e., the obturator was removed from the cannula and then reinserted so that rats were adapted to the procedure of icv injection. After adaptation, cannula placement was assessed by examining water intake in response to icv angiotensin II. Rats were deprived of water for 1 hour. They then received icv injection of 50 ng angiotensin II in 5 μl of saline and were allowed 30 min access to water. ICV injection was made with a Gilmont microliter syringe attached to polyethylene tubing and a 30-gauge stainless steel injector (Plastics One). The tip of the injector extended 1 mm past the tip of guide cannula. The criterion for correct cannula placement was water intake of 5 ml in excess of that consumed by vehicle-treated rats.

After cannula placement assessments, rats were adapted to a feeding schedule in which regular chow was removed from the cages 2 hours before lights off and returned to the cages just before dark. After habituation, rats with correct cannula placements were weight-matched and randomly divided into four groups receiving icv injection of 0, 1, 5, or 10 μg of TTR (Sigma-Aldrich Corp., St. Louis, MO) in 5 μl of saline. Injections were given in a random order right before lights out. After injection, chow was returned immediately and food intake was examined at 0.5, 1, 2, 4, and 22 hours later. Body weight change was recorded 22 hours post injection. Feeding test was repeated in a counterbalanced design so that each rat received icv saline and at least two different doses of TTR. Rats were allowed to recover for 5–7 days between each treatment. After feeding test, rats were randomly re-assigned to a saline control or TTR treatment (10 μg). ICV injection was given similarly just before lights-off, but chow was not returned after injection. One hour later, rats were euthanized, and brains were harvested for subsequent determinations of NPY levels in the DMH, the ARC, and the paraventricular nucleus (PVN) using Western blot analysis.

An additional cohort of rats equipped with lateral ventricular cannulas was used for determining whether TTR-induced reductions of NPY levels and food intake can be prevented by pre-treatment with the AMPK activator. Rats were first given an icv injection of 3 μl of saline solution (NS) or 300 μg of AICAR in 3 μl of saline (AICAR) at 1 hour before lights-off. One hour later, half of the rats from each group received 5 μl of saline and the other half received 10 μg of TTR in 5 μl of saline. Chow was returned immediately after the second injection, and food intake was measured at 0.5, 1, 2, 4, and 22 hours after the second injection. After 7 days for recovery, rats were treated again as above in a random order, but chow was not returned. One hour after the second injection, rats were euthanized and brains were harvested for subsequent determinations of NPY levels in the PVN using Western blot analysis.

### Conditioned taste aversion (CTA) test

Sprague-Dawley rats were individually housed and unilaterally implanted with lateral ventricular cannulas as above. Rats were adapted to a water-deprived schedule in which water was available for 30 min at 0900 hours, followed by 1 hour of rehydration at 1200 hours. Chow was always available except during the two periods of 30-min and 1-hour water access. Water intake was recorded. A CTA test was conducted after water intake stabilized (∼8 ml in 30 min water access) for at least 3 days. At the time of conditioning (day one), rats were randomly divided into three groups (*n* = 5–6). Following 30-min access to 0.15% saccharin in water at 0900 hours, one group of 5 rats received 5 μl of icv saline and ip injection of saline (20 ml/kg); the second group of 6 rats received icv TTR (10 μg in 5 μl of saline) and ip saline (20 ml/kg); and the third group of 5 rats received 5 μl of icv saline and ip injection of 0.15 M lithium chloride (LiCl, 20 ml/kg) as a positive control. After treatment, rats had 1 hour of rehydration at 1200 hours. On day two (a rest day), rats were maintained on the same water-drinking schedule as before. At test day three, rats were given 30-min access to two bottles of solutions (one containing water and the other containing 0.15% saccharin in water) at 0900 hours, followed by 1 hour of rehydration at 1200 hours. Intakes of both solutions were recorded.

### Primary hypothalamic neuron cultures

Primary hypothalamic neurons were prepared from Sprague-Dawley rat embryos on the 18^th^ day of gestation (Charles River Laboratories) as previously described^39^. Briefly, hypothalami were dissected and dissociated by incubation in 0.125% trypsin solution. Tissues were then triturated by repeated pipetting in DMEM culture medium containing 10% horse serum (Invitrogen, Carlsbad, CA). Cells were plated at 4 × 10^5 ^cells per 35-mm tissue culture dish and cultured in an incubator with 5% CO_2 _and 95% humidity at 37 °C for 4 hours. Medium was replaced with neurobasal medium supplemented with B27, 0.5 mM L-glutamine, and 1% pencillin-streptomycin (Invitrogen). On day 4, cytosine arabinoside furanoside (1 μM, Sigma-Aldrich) was added for inhibiting non-neuronal cell proliferation. Cells were assayed on days 8–10. To test the effect of TTR on NPY contents, cells were treated with increasing concentrations of TTR (0, 0.5, 1.0, 5.0, and 10.0 μg/ml) for 2 hours or 5.0 μg/ml of TTR at multiple time points (0, 15, 30, 60, 120 min, and 24 hours). At the end of treatment, cells were lysed for determinations of NPY levels using Western blot analyses. To test whether TTR-induced reduction of NPY levels can be prevented by pre-treatment with the AMPK activator, cells were pre-treated with either 20 μM AICAR or PBS 1 hour before addition of 10 μg/ml TTR. Two hours later, cells were harvested and lysed for determinations of NPY levels using Western blot analyses.

### Western blot

Tissues were lysed with Thermo Scientific Pierce T-PER Tissue Protein Extraction Reagent (Thermo Scientific., St. Louis, MO, USA) containing cOmplete ULTRA Protease Inhibitor and PhosSTOP Phosphatase Inhibitor Cocktails (Roche, Branchburg, NJ, USA). Proteins were separated by 4 ~ 12% SDS-PAGE gel electrophoresis and transferred to an Immun-Blot PVDF Membrane. The membrane was then incubated with specific primary antibodies, i.e., rabbit anti-TTR antibody (1:1000 dilution; ABBIOTEC., San Diego, CA, USA) or rabbit anti-NPY antibody (1:1000 dilution; ImmunoStar., Hudson, USA), followed by incubation with horseradish peroxidase-labeled goat anti-rabbit secondary antibody (Sigma-Aldrich), and detected by the SuperSignal West Pico Chemiluminescent Substrate Kit (Thermo Scientific). Anti-β-actin antibody was used for loading controls.

### Quantitative real-time RT-PCR

Total RNA was extracted from each sample with Trizol reagent (Invitrogen) according to the manufacturer’s protocols. Two-step real-time RT-PCR was performed for determinations of gene expression. One microgram of total RNA was reverse-transcribed into first-strand cDNA using the RevertAid First Strand cDNA Synthesis Kits (FERMENTAS, INC.; Glen Burnie, MD), and the resulting cDNA product was then quantified using iQ SYBR Green Supermix Kit (Bio-Rad Laboratories; Hercules, CA) on iQ5 Multicolor Real-Time PCR Detection System (Bio-Rad Laboratories). Beta-actin was used as an internal control for quantification of individual mRNAs. The following primer sets were used: *Ttr*, forward primer: 5′-atggtcaaagtcctggatgc-3′ and reverse primer: 5′-gccaagagccttccagtatg-3′; and β-actin, forward primer, 5′-tgtcaccaactgggacgata-3′, and reverse primer, 5′-gatggctacgtacatggct-3′.

### Chronic central infusion of TTR

Male OLETF and lean Long-Evans Tokushima Otsuka (LETO) rats (Hoshino Laboratory Animals, Inc.) were individually housed and unilaterally implanted with lateral ventricular cannulas as above. At 18 weeks of age, OLETF rats became obese as reported previously^10^,^12^, and were randomly divided into two groups: one group was chronically infused with icv saline and the other group was infused with icv TTR (10 g/day) via a mini-osmotic pump (0.5 μl/hr, Alzet model 2002) for 8 days. One group of LETO rats was infused with icv saline as a lean control group. At the beginning of infusion, animals were under light isoflurane anesthesia with sterile operative conditions, the mini-osmotic pump containing either saline or TTR was inserted subcutaneously between the scapulae, connected to the cannula via 5-cm-long polyethylene tubing under the skin, and the skin incision was closed with sutures. Food intake and body weight were recorded daily. Eight days later, rats were euthanized and data were analyzed.

### Statistical analysis

All values are presented as means ± SEM. Data were analyzed using StatSoft Statistica-7 software (StatSoft, Tulsa, OK). All ANOVAs were followed by pairwise multiple Fisher’s LSD comparisons. A value of *P* < 0.05 was considered to be a statistically significant difference.

## Additional Information

**How to cite this article**: Zheng, F. *et al*. Central transthyretin acts to decrease food intake and body weight. *Sci. Rep*. **6**, 24238; doi: 10.1038/srep24238 (2016).

## Figures and Tables

**Table 1:** Genes showing altered expression (>2-fold) in the dorsomedial hypothalamus (DMH) by running activity.

Change (Fold)	Gene Name & Description
10.35	transthyretin
3.536	PC4 and SFRS1 interacting protein 2
3.498	AT005664 rat brain Rattus norvegicus cDNA clone 73-P7-1(37), mRNA sequence
3.386	Similar to macrophage migration inhibitory factor (LOC364225), mRNA
2.875	Similar to leucine aminopeptidase (LOC289668), mRNA
2.793	phosphatidylinositol 3-kinase, catalytic, alpha polypeptide
2.707	Similar to RIKEN cDNA 4832412O06 gene (LOC362800), mRNA
2.42	UI-R-A0-am-c-12-0-UI.s1 UI-R-A0 Rattus norvegicus cDNA clone UI-R-A0-am-c-12-0-UI 3′
2.402	N-sulfotransferase
2.302	suppressor of cdc 2 (cdc13) with RNA binding motif 2 (LOC362138), mRNA
2.283	Transcribed sequences
2.276	Transcribed sequence with strong similarity to protein ref:NP_116253.1 (H.sapiens)
2.276	UI-R-AC1-xs-c-04-0-UI.s1 UI-R-AC1 Rattus norvegicus cDNA clone UI-R-AC1-xs-c-04-0-UI 3′
2.253	Transcribed sequences
2.252	Transcribed sequences
2.236	Transcribed sequence with weak similarity to protein pir:B28096 (H.sapiens)
2.224	Transcribed sequences
2.196	UI-R-BT1-bkl-h-06-0-UI.s1 UI-R-BT1 Rattus norvegicus cDNA clone UI-R-BT1-bkl-h-06-0-UI 3′
2.172	Transcribed sequences
2.15	v-raf murine sarcoma 3611 viral oncogene homolog 1
2.131	Similar to RIKEN cDNA 1110037D14 gene (LOC289469), mRNA
2.127	Transcribed sequences
2.118	Transcribed sequences
2.116	Similar to sorting nexin 10 (LOC297096), mRNA
2.093	Transcribed sequences
2.079	Similar to RIKEN cDNA 5330414D10 (LOC311726), mRNA
2.074	UI-R-CT0-bty-g-05-0-UI.s1 UI-R-CT0 Rattus norvegicus cDNA clone UI-R-CT0-bty-g-05-0-UI 3′
2.071	Similar to Nap1l2 (LOC317247), mRNA
2.065	Similar to RIKEN cDNA 1110055L24 (LOC295629), mRNA
2.057	Similar to proteasome 26S ATPase subunit 6 (LOC289990), mRNA
2.054	cAMP-regulated guanine nucleotide exchange factor II
2.052	pyroglutamyl-peptidase I
2.038	Similar to hypothetical protein DKFZp547C176 (LOC315670), mRNA
2.033	Transcribed sequence with strong similarity to protein ref:NP_062424.1 (M.musculus)
2.018	UI-R-BO0-aic-c-02-0-UI.s1 UI-R-BO0 Rattus norvegicus cDNA clone UI-R-BO0-aic-c-02-0-UI 3′
0.481	hepatocyte nuclear factor 3, beta

Microarray analysis of rat genome shows that using criterion of a 2-fold change, 36 out of the total 31,099 genes were identified in the DMH of rats with voluntary access to running wheels for 3 days relative to sedentary rats. *n* = 6 rats per group.
